# Dysregulation of miRNA Expression in Cancer Associated Fibroblasts (CAFs) and Its Consequences on the Tumor Microenvironment

**DOI:** 10.3390/cancers9060054

**Published:** 2017-05-24

**Authors:** Maren Schoepp, Anda Jana Ströse, Jörg Haier

**Affiliations:** 1 Comprehensive Cancer Center Münster (CCCM), University Hospital Münster, 48149 Münster, Germany; maren.schoepp@ukmuenster.de (M.S.); anda.stroese@ukmuenster.de (A.J.S.); 2 Nordakademie University of Applied Sciences, Köllner Chaussee 11, 25337 Elmshorn, Germany

**Keywords:** microRNA, cancer associated fibroblasts, cell-cell communication, transformation, epigenetic

## Abstract

The tumor microenvironment, including cancer-associated fibroblasts (CAF), has developed as an important target for understanding tumor progression, clinical prognosis and treatment responses of cancer. Cancer cells appear to transform normal fibroblasts (NF) into CAFs involving direct cell-cell communication and epigenetic regulations. This review summarizes the current understanding on miR involvement in cancer cell—tumor environment/stroma communication, transformation of NFs into CAFs, their involved targets and signaling pathways in these interactions; and clinical relevance of CAF-related miR expression profiles. There is evidence that miRs have very similar roles in activating hepatic (HSC) and pancreatic stellate cells (PSC) as part of precancerous fibrotic diseases. In summary, deregulated miRs affect various intracellular functional complexes, such as transcriptional factors, extracellular matrix, cytoskeleton, EMT/MET regulation, soluble factors, tyrosine kinase and G-protein signaling, apoptosis and cell cycle & differentiation, but also formation and composition of the extracellular microenvironment. These processes result in the clinical appearance of desmoplasia involving CAFs and fibrosis characterized by deregulated stellate cells. In addition, modulated release of soluble factors can act as (auto)activating feedback loop for transition of NFs into their pathological counterparts. Furthermore, epigenetic communication between CAFs and cancer cells may confer to cancer specific functional readouts and transition of NF. MiR related epigenetic regulation with many similarities should be considered as key factor in development of cancer and fibrosis specific environment.

## 1. Introduction

Over the years the tumor microenvironment has developed as an important target for understanding tumor progression, clinical prognosis and treatment responses of cancer, such as for chemotherapy and radiation. General understanding of a dynamic relationship between the expanding tumor and the host surrounding tissue is advancing. Cancer-associated fibroblasts (CAF) are a major cellular constituent of this tumor stroma, but little is known how cancer cells transform normal fibroblasts (NF) into CAFs and about the cell-cell communication between cancer cells and CAFs. These cells and their interactions seem to be relevant for various cancer related phenomena, but also for resistance against treatment modalities [1]. For example, CAFs can promote tumorigenesis, growth, invasion and metastasis of cancer, whereas NFs are thought to suppress tumor progression [2]. As one key event CAF activation appears to induce alternative production and secretion of extracellular matrix (ECM) proteins resulting in ECM remodeling and cancer cell invasion [3]. Resulting desmoplasia is not only attributed to CAFs but also to surrounding stellate cells, which are believed to play critical roles in conferring cancer aggressiveness.

Although it is fully accepted that microRNA (miRs) are deregulated in human cancers, we are only at the beginning of elucidating whether miR expression and function in resident fibroblasts of the tumor microenvironment are affected by their interactions with cancer cells. There is increasing evidence that miRs are involved in the transformation from NFs into CAFs and that vice versa, miRs released from CAFs can affect various characteristics of cancer cells. Collectively, published results suggest a crosstalk between CAFs and cancer cells, which in part may confer increased aggressiveness to the tumors [4].

MiRs are small noncoding RNA molecules that negatively regulate gene expression at a post-transcriptional level. Their target genes are known to affect cell differentiation, adhesion, migration, proliferation, secretion and cell-cell interaction, among others. It has also been proposed that miRs can reprogram various somatic cells to become pluripotent stem cells [5]. MiRs frequently occur in clusters at a short inter-miR distance [6] and are usually combined by a single promotor/transcription region for all cluster members. They have to be differentiated from miR families where sequence homology is the major determinant. Clustering appears to be an important way of evolutionary spreading of miR genes throughout the human genome and many clusters have a significant degree of evolutionary conservation [7], indicating that miR clustering is important for their biological role due to combined regulation of their expression [8].

The aim of this review was to summarize the current understanding on: (a) how miRs are involved in cancer cell—tumor environment/stroma communication, specifically the transformation of NFs into CAFs, their involved targets and signaling pathways in these interactions; and (b) whether CAF-related miR expression profiles are clinically relevant. By reviewing the literature we also found evidence that miRs have very similar roles in the activation of hepatic (HSC) and pancreatic stellate cells (PSC) which are also specifically activated fibroblasts being part of precancerous fibrotic diseases, like pancreatic and hepatic fibrosis. HSC and PSC are major determinants of the well-known phenomenon desmoplasia and are considered as significant part of the precancerous microenvironment in fibrotic tissue, esp. in these two organs. Therefore, miR-related similarities and differences related to tumor-related cellular dysfunctions between the various types of activated fibroblasts were included in this review.

## 2. Methods

A PubMed search was performed using combinations of the following keywords: miR, microRNA, cancer associated fibroblasts, cancer and fibroblasts, hepatic stellate cells (HSC) or pancreatic stellate cells (PSC). Articles that met the criteria included:

- ([mir] OR [miR] OR [microRNA]) AND [cancer] AND ([caf] OR [cancer associated fibroblasts]) = 184;
- ([mir] OR [miR] OR [microRNA]) AND [cancer] AND ([hsc] OR [hepatic stellate cells]) = 64;
- ([mir] OR [miR] OR [microRNA]) AND [cancer] AND ([psc] OR [pancreatic stellate cells]) = 15.

Relevant publications were identified by screening the abstracts and the text of the entire published paper as necessary. Reference lists from relevant articles were also searched for additional literature. Data were extracted from the article, or if only available from the abstracts. This method was applied until the beginning of May 2016 and has been updated in January 2017.

A narrative review of relevant literature was conducted to identify and summarize evidence that miRs play a pivotal role in the communication between cancer cells and environmental fibroblasts. Due to clinical and functional similarities of fibroblasts in cancers and precancerous lesions, pancreatic and hepatic fibrosis were included in this search. Specific focus was given towards cluster organization of the reported miRs.

## 3. Clinical Relevance of miR in CAF

Very few data are available on deregulated miR-expression in CAFs from clinical specimens. Up- and downregulation of miRs has been found comparing normal or tumor-adjacent fibroblasts. For example, in ovarian CAFs, miR-31 and miR-214 were downregulated, whereas miR-155 was upregulated [5]. In contrast, expression of miR-31 in CAFs was higher than in normal colorectal fibroblasts [9]. In endometrial cancer miR-148a [10] and miR-31 [11] were downregulated. MiR-26b was downregulated and miR-92 was upregulated in CAFs from estrogen receptor (ER)-positive breast cancers [12,13]. Downregulation of miR-200 family members in activated CAFs has been found in breast [3,14], gastric [15] and pancreatic cancer [16]. Interestingly, a reciprocal correlation of miR-200 and its putative target expression (SIP1 and E-cadherin) compared between pancreatic CAFs and cancer specimens was reported [16]. In addition, miR-21 was frequently upregulated in CAFs in various entities [17,18]. Moreover, expression of this miR in stellate cells derived from normal pancreas was substantially lower when compared to PSCs or CAF cells [4]. Specific upregulation of miR-409-3p and miR-409-5p was found in prostate cancer stromal tissue specimens [19], whereas miR-15 and miR-16 were downregulated in fibroblasts surrounding prostate tumors [20]. Additional mixed patterns were found by array technologies in breast [10,14], lung [21] and prostate [22] cancer specimens in very small patient cohorts, but the evidence for reproducible cancer specificity of such signatures is still very limited. These genome-wide screenings provided potentially deregulated miRNAs in CAFs from different entities (upregulated: miR-221-5p, miR-31-3p, miR-221-3p; and reduced: miR-205, miR-200b, miR-200c, miR-141, miR-101, miR-342-3p, let-7g, miR-26b) (Table 1). Interestingly, contradictory results between clinical and experimental samples have been reported, such as for miR-106b (compare Table 2). These different observations of dysregulated miR has also been observed in other context for which the authors provided the model of regulators freedom for miR regulation (see review in ref. [8]).

Prognostic impact of altered miR expression in CAFs in clinical cohorts has been observed for patients with breast cancer (miR-26b) [12], gastric cancer (miR-106b [23], miR-143 [24], miR-145 [25], miR-200b [15]), esophageal carcinoma (miR-21 [17], miR-27a/b [26]) and pancreatic adenocarcinoma (miR-21 [17]). Furthermore, miR-21 expression in CAFs was elevated in colorectal cancer specimens compared to fibroblasts in colonic polyps [27] and it was associated with decreased overall survival in pancreatic cancer patients who received 5-FU, but not gemcitabine. [18] It has also been reported that miR-27a/b are involved in resistance to chemotherapy in esophageal cancer through miR-27a/b-induced transformation of NFs into CAFs [26].

Clinical expression data for PSCs have not been published yet, but for HSCs and liver fibrosis several investigations are available. The hepatic contents of miR-21 [28,29], miR-33a [30] and miR-200b [31] were significantly increased in liver specimens from human patients with liver fibrosis as compared to normal patients. Upregulation was also identified for miR-199a-5p/199a-3p and miR-221/222 in hepatitis C induced liver fibrosis in a fibrosis progression-dependent manner [32]. Members of the miR-17-92 cluster (19a, 19b, 92a) [33], miR-29, miR-133, miR-193 and miR-30c [34,35] were observed to be specifically downregulated in human liver fibrosis and HSC, while they showed a reciprocal expression pattern after recovery from liver fibrosis. Reduced expression of miR-144 was correlated with elevated HSC-specific expression of transforming growth factor-β1 (TGF-β1) and expression of α-SMA in fibrotic liver tissues. In contrast, miR-200c did not show comparable differences and correlations [36]. Hepatic expression levels of miR-199a, miR-199a*, miR-200a, and miR-200b were positively associated with progression of liver fibrosis [31,37].

In liquid biopsies, levels of miR-17-5p [38], miR-21 [28], miR-33a [30] and miR-181b but not miR-181a expression [39] were higher in fibrotic than in normal patients. In addition, miR-133a serum levels were increased in patients with chronic liver disease and indicative for the presence and progression of liver cirrhosis [35].

Overall, the body of evidence for clinically useful miR expression patterns in CAFs as prognostic or predictive markers is not yet sufficient for any recommendation into clinical applications. In addition, a systematic approach for identification of the relevant miR expression profiles has not been done thoroughly and, therefore an overall picture for patients is not available yet.

Most promising candidates for future clinical use appear to be miR-21, miR-31 and the miR-200 family. Furthermore, a number of miRs that are known for their involvement in cancer cells have been identified to also be important in patients derived CAFs suggesting a further look into their functional consequences.

## 4. Functional Consequences of miRNA Dysregulation in CAFs

Molecular mechanisms leading to transition of tissue-resident fibroblasts into CAFs, circulating bone marrow-derived fibroblast progenitors or mesenchymal stem cells are largely unknown [22]. The spectrum of miRs that have been identified as relevant regulators of this CAF transition and/or for their interactions with cancer cells is relatively limited. Except for two publications investigating squamous cell esophageal carcinomas [26,40] all data were derived from adenocarcinomas of different organs. Interestingly, the majority of these miRs belongs to miR clusters (<10 kb sequence distance between the members) with functional overlap between the various cluster members. Deregulation of miRs in fibroblasts in general appears to induce as major effects smooth muscle α-actin (α-SMA) upregulation and modulated secretion of cytokines including: increased IL-6 and CXCL12, activation of the TGF-β and inhibition of various signaling pathways, such as the PI3K-AKT pathways [2,3,21]. Various miRs interfere with multiple cellular functions and therefore be found at different point in the subsequent listing.

An overview about the miRs with altered expression in CAFs is provided in Table 2 (extended data are available in Supplemental (Table S1) showing that various miRs occur up- and downregulated even within the same tumor entity, such as miR-101, miR-200a and miR-200b. It appears quite interesting, that those miRs (except miR-101) belong to the miR-200 family, which is clustered at chromosomes 1 and 12. Similar observations have been summarized recently for the miR23~24~27 clusters in cancer cells [8].

### 4.1. EMT/MET Switch

The known functions of miR-200 family members in the EMT/MET switch (targeting ZEB1, ZEB2; E-cadherin expression) can also be found in CAFs [2]. MiR-146b inhibition is sufficient to transactivate NFs into CAFs, which promote EMT transition in breast cancer cells in a paracrine manner [41]. In a similar manner miR-148a targets genes of the WNT family, WNT1 and WNT10b with comparable cellular effects [10]. Furthermore, miR-21 overexpression was associated with enhanced ERK1 signaling and EMT in liver fibrosis via direct suppression of SPRY2 and HNF4α expression [28]. Promotion of tumor induction and EMT was also induced by ectopic expression of miR-409 (member of a very large cluster) in prostate NFs conferring a CAF-like phenotype and leading to the release of miR-409 via extracellular vesicles. This miR also enhanced tumorigenesis through repression of tumor suppressor genes such as Ras suppressor-1 (RSU-1) and stromal antigen 2 (STAG2) [19]. Additional targets were observed that are also involved in both processes, including Fli-1 (directly regulated by miR-200c), TCF12 (directly targeted by miR-141) [3], SIP1 (by promoter methylation via miR200a/b) [16] and Flt-1 (directly targeted by miR-200b) [42]. CAF exosomes and NF exosomes transfected with miRs-21, -378e, and -143 promoted the stemness and EMT phenotype of breast cancer cells [43].

### 4.2. TGF-β Signaling

A relatively large body of data has been published for miR-21 (chromosome 17q23.2) which is one of the few non-clustered miRs that have been found to be relevant in CAFs. This miR may be an important factor in “activating” NFs into CAFs and its expression is mostly confined to cancer stroma [4,17]. Via direct targeting miR-21 is a negative regulator of programmed cell death protein 4/activation protein-1 (PDCD4/AP-1) in cancer cells [27] and HSCs [29]. This appears to be based on a miR-21 feedback loop with these signaling pathways promoting fibrogenesis and the TGF-β signaling pathway underlying HSC activation [29].

MiR-145 is inducible by treatment with TGF-β leading to enhanced α-SMA expression in normal gastric fibroblasts and CAFs [25]. MiR-27a/b-transfected normal fibroblast showed α-SMA expression and increased production of TGF-β as typical characteristics of CAFs [26]. In addition, miR-126 can support TNFα induced TGF-β1 expression [44]. MiR-205 has been observed as most downregulated miR in prostate cancer cells upon CAF stimulation due to direct transcriptional repression by HIF-1α, a known redox-sensitive transcription factor [45]. In addition, miR-127 is the best described member of a large cluster of miRs on chromosome 14 that also act as key modulators of TGF-related cellular senescence by targeting critical regulators of the senescence pathways. This miR is upregulated in senescent fibroblasts and may function as tumor suppressor and inhibitor of breast cancer cell proliferation by modulating the BCL-6 oncogene [46].

Functional data for members of the three miR-17-92 clusters in cancer are almost lacking. For fibrotic progression, however, various investigations showed the importance of these cluster members for intensive interactions with TGF-β signaling. For example, HSC activation is a pivotal event in initiation and progression of hepatic fibrosis and a major contributor to collagen deposition driven by TGF-β. In animal models miR-19b showed the highest fold-change of the cluster members. Its mimic negatively regulated TGF-β signaling components including decreased TGF-β receptor II (TGF-βRII) (by direct binding) and SMAD3 reduced expression of type I collagen and blockage of TGF-β-induced expression of α1(I) and α2(I) procollagen mRNAs. Similarly, enhanced miR-17 expression was observed in rat liver fibrosis. Its inhibition also led to suppression of HSC proliferation induced by TGF-β1 without affecting cellular apoptosis, but with significant association of type I collagen and α-SMA expression in HSC [38].

Comparable to the tumor environment expression levels of fibrosis related genes in HSCs were increased by overexpression of miR-200 family members (miR-200a, and 200b) [37]. For example, miR-200a was decreased in TGF-β1-induced HSC activation and induced liver fibrosis. Overexpression of miR-200a in HSCs inhibited α-SMA activity and proliferation. In addition, β-catenin and TGF-β2 were confirmed as two functional downstream targets of miR-200a in the fibrotic framework [47]. Divergent roles of miR-181 cluster members in HSCs have been reported. MiR-181b but not miR-181a could promote HSC proliferation induced by TGF-β1 through regulation of cell cycle (targeting p27^Kip1^) [39]. Similarly, the X-chromosomal miR-221/222 is increased in liver fibrosis models (stimulated by TGF-α or TNF-α). MiR-222 can bind to CDKN1B in HSCs and its induction can be suppressed by NF-κB inhibitor [32].

Downregulated miR-101 appears to increase CAF-promoted vascular mimicry formation in vitro and in vivo. Gain- and loss-of-function analyses revealed that the miR-101-TGF-β/SDF1-VE-cadherin/MMP2/LAMC2 network mediates this formation of vascular-like channels [48]. Members of the miR-101 family (miR-101a/b) can act as suppressors of TGFβ signaling by directly targeting TGF-βRI and its transcriptional activator Kruppel-like factor 6 (KLF6) during liver fibrogenesis where these miRs are reduced in activated HSCs. Meanwhile, upregulation of TGF-βRI/KLF6 was observed in the fibrotic liver [49].

### 4.3. Extracellular Matrix, Migration and Invasion

MiR-122 overexpression also led to decreased collagen maturation and ECM production [50] whereas miR-126 [44] and miR-17-92 cluster members [38] induced type 1 collagen expression. Reduced expression of miR-335 during HSC activation promotes their cell migration via targeting tenascin-C and enhanced expression of α-SMA and type 1 collagen [51]. Furthermore, miR-19b blunted the activated HSC phenotype by morphological assessment and decreased α-SMA expression [33]. In HSCs another cluster member, miR-133a, is downregulated by TGF-β with subsequent stimulated expression of collagens [35]. The expression levels of fibrosis related genes (TIMP-1, MMP13, α1-procollagen) in HSCs were increased by overexpression of other members of the miR-214 cluster (miR-199a, 199a*) [37]. Moreover, miR-200b appears to enhance expression of matrix metalloproteinase-2 (MMP-2), which may increase the migration of HSCs during liver fibrosis progression [31]. MiR-31 may also mediate liver fibrosis by promoting HSC activation and enhancing MMP-2 expression. Functionally this appears to be mediated via FIH1, a suppressor of hypoxia-inducible factor (HIF-1) [52].

Among others, miR-200 family members were identified as potential direct mediators of NF reprogramming into CAFs and of ECM remodeling. NFs with downregulated miR-200s displayed the traits of activated CAFs, including accelerated migration and invasion [3]. Overexpression of miR-200b can enhance migration and proliferation of fibrotic HSCs; this is accompanied by stimulated phosphorylation of Akt, a downstream effector of phosphatidyl-inositol 3-Kinase (PI3K). Within this PI3K/Akt pathway FOG2 is an additional target of miR-200b that directly binds to p85α inhibiting activation of the signaling. In fibroblasts with amplification of miR-92 expression an enhanced invasive capacity of breast cancer cells was reported [13]. CAFs with downregulated miR-106b could significantly inhibit gastric cancer cell migration and invasion by targeting PTEN [23]. Similarly, miR-335 interferes with a senescence-associated secretory phenotype of CAF via modulating PTEN [53]. In addition, miR-21 can play a role in expression and activity of MMP2 and HSC activation through PTEN/Akt pathway [54]. Furthermore, miR-7 in NFs significantly increased the migration activity and growth rates of cancer cells in co-culture experiments which seems to be mainly mediated by the RASSF2-PAR-4 axis [55].

Intercellular communication between ER-positive breast cancer cells and CAFs reduced miR-26b expression in CAF but enhanced cancer cell migration and invasion. Pathway analyses of differentially expressed proteins revealed that glycolysis/TCA cycle and cytoskeletal regulation by Rho GTPases are downstream of miR-26b. In addition, its targets (TNKS1BP1, CPSF7, COL12A1) were identified and their expression in cancer stroma was shown to be significantly associated with breast cancer recurrence [12]. Both, miRNA-150 and miRNA-194 inhibit HSC activation and ECM production, at least in part, via inhibition of c-MyB and Rac-1 expression [56].

In fibrotic stromal reaction of pancreatic cancer [57] and in liver fibrosis [58] members of the miR-29 cluster (miR-29a) are significantly decreased. This loss influences the activation of CAFs/PSCs/HSCs and is correlated with increase in extracellular matrix (ECM) deposition including type I collagen-α1. This profibrogenic phenotype is likely caused by inhibition of HDAC4 function including histone acetylation and mediated by SMAD3 dependent TGF-β1 activation. In contrast to other family members miR-146a (chromosome 5) has been found downregulated in liver fibrotic tissues [59] and in HSCs in response to TGF-β1 or TNF-α stimulation with subsequent α-SMA/type 1 collagen expression and attenuated HSC apoptosis via direct targeting of TIMP-3 [60], Wnt1 and Wnt5a [58]. In vitro, miR-210 was identified as upregulated in and secreted by PSCs inducing activated ERK and Akt, but not hypoxia-inducible factor-1α (HIF-1α) pathway in effector cancer cells. Subsequently, this stimulated migration, expression of vimentin and Snail-1, and blocked membrane-associated expression of β-catenin [61].

### 4.4. Soluble Factors

Interactions between CAFs and cancer cells by soluble factors appears to induce most of the transcriptional changes characteristic for patient-derived CAFs. For example, miR-15/miR-16 cluster members (chromosomes 3 & 13) and their correlated growth factor targets, such as fibroblast growth factor-2 (FGF-2) and its receptor FGFR-1, seem to promote tumor expansion and invasiveness through the concurrent activity on stromal and cancer cells. Similarly, miR-483 members act together to target two pro-fibrosis factors, platelet-derived growth factor B (PDGF-B) and TIMP-2, which can suppress activation of HSCs and their overexpression can reduce liver fibrosis in vivo [62]. In the opposite regulatory direction, among a panel of soluble growth factors only TGF-β remarkably increased miR-31 expression in these cells by direct binding of SMAD3 to miR-31’s promoter suggesting regulatory counteraction of both molecules [52]. Treatment of HSCs with PDGF-B stimulated a1(I) collagen mRNA synthesis and the protein expression of α-SMA in a miR-21 dependent manner, which both are characteristics of HSC activation and simultaneously increased miR-21 expression [54]. This suggests feedback loops of activation.

Comparably, miR-133b (member of the miR-133 cluster) interferes with paracrine communication of CAFs by interleukin-6 (IL-6) that can promote tumor progression [22]. Mediated by miR-146b (chromosome 10) and by repression of IL-6 secretion the tumor suppressor p16INK4A protein inhibits the pro-carcinogenic effects of breast stromal fibroblasts. In addition, miR-205 blocks tumor-driven activation of surrounding CAFs by reducing pro-inflammatory cytokine secretion and vice versa prevents CAF-induced EMT in cancer cells in vitro [45]. The miR-193b/-365a cluster is also involved in cytokine related cell-cell communication. TGF-β-dependent downregulation has been identified involving Snail-1, an important regulator of extracellular matrix in HSCs [34]. Suppressed NF-κB dependent p65 activation inhibits miR-365 expression with resulting increased IL-6 secretion within the cancer environment [63].

Furthermore, the miR-reprogrammed normal fibroblasts and patient-derived CAFs shared a large number of upregulated genes highly enriched in chemokines, which are known to be important for CAF function. The most highly upregulated chemokine, CCL5, (C-C motif ligand 5) was found to be a direct target of miR-214 [5]. Similarly, estradiol (E2) as another soluble environmental factor and a major determinant of gender-based differences in the development of hepatic fibrosis was modulated by miR-19b in regulation of HSC proliferation via direct interference with GABA (B) receptor GRB2 [64].

The role of other forms of miR-involvement in cell-cell communication has been recently introduced. Pancreatic cancer cells can reprogram adjacent NFs into CAFs by means of secreted microvesicles containing miR-155 likely to be considered as exosomal miR transfer [65]. Comparably, miR-21 release from fibroblasts appears to influence migration and invasion capacity of cancer cells [27].

### 4.5. Cell Cycle and Proliferation

Downregulated miR-15a/miR-16-1 in CAFs can promote prostate cancer growth, progression and cancer cell survival, proliferation and migration. Their reconstitution impaired tumor supportive capability of these modified stromal cells [20]. In contrast, members of the paralog cluster (miR-15b/miR-16-2) seem to induce apoptosis in activated PSC (targeting Bcl-2 [66]) and HSC (cyclin D1 [67]) and their decreased expression can support pancreatic and liver fibrosis, respectively. Downregulated miR-146a also attenuated HSC apoptosis via direct targeting of SMAD4 [68].

The synthesis pathways of various ECM proteins, especially collagens, is targeted by miRs in fibroblasts and thereby enhancing the fibrotic phenotypes. For example, in vitro and in a mouse fibrosis model miR-122 expression was reduced in activated HSCs supporting their proliferation which appears to be mediated by direct targeting of prolyl 4-hydroxylase subunit alpha-1 (P4HA1). Binding activity of CCAAT/enhancer binding protein alpha (C/EBPα) to the miR-122 promoter region was reduced in these cells [49]. Furthermore, one member of the miR-33 family (miR-33a), which is located in intronic regions within protein-coding genes for Sterol regulatory element-binding proteins (SREBP-2 and SREBP-1), is highly expressed during TGF-β1 induced activation of the PI3K/Akt pathway in HSCs. This appears to result in expression of type 1 collagen (Col1A1) and α-SMA due to direct targeting of peroxisome proliferator activated receptor-alpha (PPARα) [30]. Similarly, miR-34a has not been described in CAFs yet, but in activated HSCs and liver fibrosis it was found to be upregulated, regulating a plethora of target proteins involved in the cell cycle, apoptosis, differentiation and cellular development, such as upregulated PPARγ and downregulated α-SMA [69]. PPARγ maintains HSCs in a quiescent state, and its downregulation induces HSC activation. This appears to be related to TGF-β1 induced enhanced expression of various members of the miR-130 precursor family (miR-130a, miR-130b, miR-301a), miR-27b and miR-340 in liver fibrosis. Overexpression of miR-130a and miR-130b enhanced cell proliferation involving Runx3 and upregulation of ECM genes. [70] Such quiescent HSC phenotype can also be stimulated by overexpression of miR23~24~27 cluster members (likely via targeting retinoid X receptor) [19] and miR-146a with decreased cell proliferation [68].

Cellular senescence acts as a barrier to cancer progression, and non-clustered miR-22 is thought to potentially regulate senescence due to growth suppression in human normal and cancer cells. Its knockdown in pre-senescent fibroblasts decreased cell size, motility and invasion. This seems to be mediated by direct targeting of CDK6, SIRT1, and Sp1, genes involved in the senescence program [71]. Upregulation of miR-9a and downregulation of its target SIRT1 were observed in fibrotic liver models. Overexpression of SIRT1 could partially abrogate the miR-9a mediated suppression of HSCs proliferation, migration and activation [72].

A number of other transcription factors have been identified as miR-targets in fibroblasts. For example, miR-200a can regulate the Keap1/Nrf2 pathway in HSCs and hepatic fibrosis through the association of Nrf2 with Keap1, which results in cytoplasmic Nrf2 degradation and interference with induction of genes that encode antioxidant enzymes, such as NQO1 [73]. In addition, in CAFs miR-31 is among the most downregulated miRs that directly targets the homeobox gene SATB2 relating to chromatin remodeling and regulating of gene expression during CAF transition [1]. However, in PSC upon activation, this miR was upregulated, among others [74]. In HSCs and PSCs reduced miR-126 was observed [74] promoting the inhibition of NF-κB activation by upregulation of IκBα protein expression.

## 5. MiR-Targeted Readouts for Cellular Functions

Since miR regulation is not limited to effector chains, linear regulation models and classical feedback loops, we summarize the effects of miR-dysregulation on complex cellular functions in the targeted CAFs. Importantly, the expression of many miRs that are deregulated in fibroblasts associated with pathological conditions appear to be regulated themselves by soluble factors, including TGF-β as main regulator, but also by other growth factors, IL-6, TNF-α and several chemokines. In addition, few proteins (c/EBPα, SIRT1) were identified that bind to miR promotor regions hereby modulating miR transcription. Furthermore, feedback loops have been described for TGF-α and IL-6 that appear to modulate expression of a large variety of miRs.

**Figure 1:**
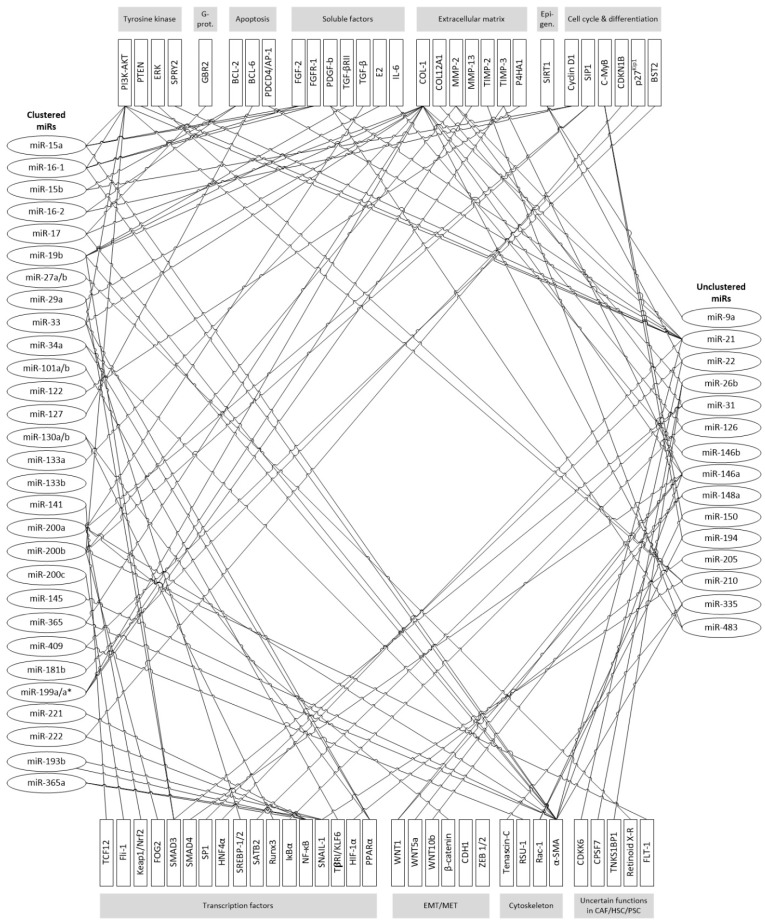
Currently known miR target structures in CAF/HSC/PSC. Targets are grouped according to main cellular functions, miRs are ordered in clusters (left), unclustered miRs are grouped on the right side.

Most miR targets that have been found in CAFs/HSCs/PSCs can be grouped into various functional complexes. These include transcriptional factors, extracellular matrix, cytoskeleton, EMT/MET regulation, soluble factors, tyrosine kinase and G-protein signaling, apoptosis and cell cycle & differentiation (Figure 1). However, as known for many miRs also in CAFs and related cell types multiple (N-to-N) relationships can be preferably found resulting in a large network of interactions. Considering the phenomenological appearance of desmoplastic reactions and fibrosis it is not surprising, that many regulatory interactions of identified miRs focus at cytoskeleton, esp. α-SMA, and ECM, esp. type 1 collagen.

The largest number of identified targets belongs to the transcriptional machinery with a high variety of interactions. This appears to be relevant for the transition of NF into their pathological counterparts with subsequent changes in the readout of many cell functions and modulated protein expression profiles in CAFs/HSCs/PSCs and their microenvironment.

**Figure 2:**
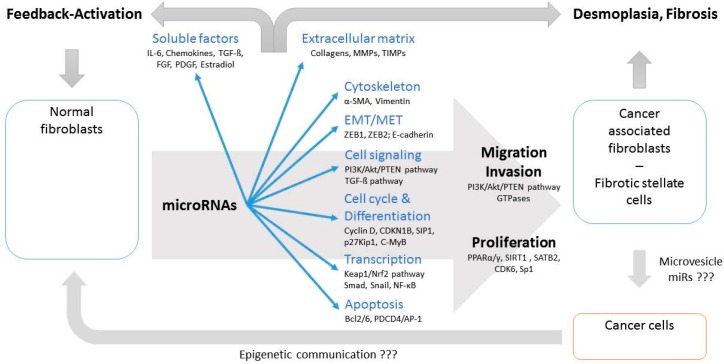
Deregulated miRs affect intracellular functional complexes in fibroblasts and formation of surrounding ECM; thus supporting transition of NFs into their pathological counterparts and development of desmoplasia and fibrosis. Modulation of soluble factors can act as (auto)activating feedback loop. Epigenetic communication between CAFs and cancer cells, such as by extracellular microvesicles, is likely, but rarely investigated. The most important targets affected by miRs within fibroblasts are added in relation to involved cellular functions. (???: insufficient evidence available)

MiR related epigenetic regulation should therefore be considered as key factor in the development of cancer and fibrosis specific environment and both transitions have many epigenetic similarities. Although many deregulated miRs in CAFs/HSCs/PSCs belong to miR clusters functionally different consequences between clustered and non-clustered miRs cannot be attributed according to the currently available literature (Table 3). However, due to the fact that about two third of the identified miRs belong to clusters it seems to be likely that conjoint deregulation of clustered miRs might play important roles in transforming NF into their pathological counterparts. In summary, deregulated miRs affect various intracellular functional complexes, but also formation and composition of the extracellular microenvironment. These processes result in the clinical appearance of desmoplasia involving CAFs and fibrosis characterized by deregulated stellate cells. In addition, modulated release of soluble factors can act as (auto)activating feedback loop for transition of NFs into their pathological counterparts. Furthermore, epigenetic communication between CAFs and cancer cells, for example via extracellular microvesicles, may confer to cancer specific functional readouts (Figure 2).

## Figures and Tables

**Table 1:** Direction of deregulated miR expression in CAFs in clinical cohorts of various cancer entities.

Alteration in Clinical Specimens	microRNA	Cancer Entity								
		Breast	Ovarian	Endometrial	Esophageal	Gastric	Colorectal	Pancreatic	Prostate	Mixed Entities
upregulated	miR-21				↑		↑	↑		
	miR-31						↑			↑
	miR-92	↑								
	miR-221									↑
	miR-409								↑	
	miR-155		↑							
downregulated	miR-15								↓	
	miR-16								↓	
	miR-26b	↓								
	miR-31		↓	↓						
	miR-101									↓
	miR-106b					↓				
	miR-141	↓								↓
	miR-148a			↓						
	miR-200	↓_abc_*				↓_b_*		↓_ab_*		↓_bc_*
	miR-205									↓
	miR-214		↓							
	miR-342									↓
	miR-let7g									↓

* reported members of the miR-200 family are listed accordingly.

**Table 2:** Deregulated miRs in (**A**) CAFs and (**B**) HSC/PSC, consequences on cellular functions and identified target structures. MiRs that occur in clusters are highlighted with blue background.

**(A)**														
**MiR with Cluster**	**Cancer Entities**	**Regulation of miR in CAFs**	**Interaction in CAFs**	**Cellular Consequences**									**Pathway**	**Targets**
				**Migration**	**Invasion**	**Adhesion**	**Growth**	**Proliferation, Progession**	**Differentiation**	**Chemoresistance**	**Apoptosis**	**Methylation**		
15, 16	PC	↓	α-SMA ↑	↑			↑	↑					p-AKT, p-ERK ↑	Fgf-2 and its receptor Fgfr1 ↑
27a/b	EOC	↑	α-SMA ↑							↑	↓		TGF-β ↑	
92	BC	↓			↑									
106b	GC	↑	α-SMA ↑	↑	↑		↑						TGF-β ↑	PTEN
101	BC, LC	↓↑	α-SMA ↑, IL-6 ↑	↓↑	↓↑	↓		↓↑	↓		↑	↓	PI3K-AKT ↓, TGF-β ↑	CXCL 12 ↓
143, 145	GC	↑	α-SMA ↑, Collagen Typ III ↑		↑		↑	↑					TGF-β /SMAD signaling ↑	
200a, 200b	BC, LC, PaC, GC	↓↑	α-SMA ↓↑, IL-6 ↑	↓↑	↓↑	↓↑		↓↑	↓↑			↓	TGF-β ↑↓	ZEB1, ZEB2 ↑; Flt 1 ↓; SIP1
221	BC, PaC	↑	α-SMA ↑, IL-6 ↑	↑	↑					↑			TGF-β ↑	NF-κB, K-Ras ↑
214	OC	↓	cytokines ↑	↑	↑		↑							CCL5
127	BC	↓						↑					p53/p21 ↑	BCL6 oncogene ↑
133b	PC	↑	α-SMA, IL-6 ↑, Collagen 1A1					↑					TGF-β ↑	
141, 200c	BC	↓↑	α-SMA ↑, IL-6 ↑	↑	↑	↑		↑	↑			↑	TGF-β ↑	Fli-1, TCF12
342	BC	↓	α-SMA ↑, IL-6 ↑	↑	↑	↑		↑	↑			↑	TGF-β ↑	
365	BC	↓	IL-6 ↑	↑	↑								p38 MAPK ↓	NF-κB p65 ↑
409	PC	↑	α-SMA, EMT ↑, extracelluar vesikel (EV) release ↑				↑	↑						Ras suppressor 1, stromal antigen 2
**(B)**														
**MiR with Cluster**	**PSC/HSC**	**Regulation of miR in PSC/HSC**	**Interaction in Cells**	**Cellular Consequences**									**Pathway**	**Targets**
				**Migration**	**Growth**	**Development, Movement**		**Proliferation, Progession, Activation**			**Fibrosis, Apoptosis**			
15, 16	PSC	↓									↑			BCL-2 ↑
27a/b	HSC	↑						↑						retinoid X receptor alpha
17, 19b	HSC	↑↓	Collagen Type I and α-SMA ↑↓, expression of α1(I) and α2(I) procollagen in mRNAs ↑		↓			↓			↑		TGF-β 1 ↑↓	SMAD7 ↓, TGF-β 2 rezeptor and SMAD 3, GRB2
101	HSC	↑		↓				↓					TGF-β ↓	TβRI/KLF6 ↓
143	PSC	↑			↑	↑							Smad 2/4	p39 MAP kinase & extracell.-signal–regulated kinase
200a, 200b	HSC	↓↑	α-SMA , EMT process ↓	↑	↓			↓↑			↑		Wnt/β-catenin, TGF-β , PI3K/Akt ↑, Hh pathway	FOG2 ↓-regulation, Keap1/Nrf2 ↑, Gli2 ↓
221, 222	HSC, PSC	↑	α1Collagen and α-SMA ↑		↑	↑					↑		Smad 2/5, NF-κB	p40 MAP kinase & extracell.-signal–regulated kinase
214	HSC	↓	Collagen Type I ↓								↑			Cox-2 protein expression, NF-κB ↑
29a	HSC, PSC	↓↑	Collagen Type I		↑	↑		↑			↑		TGF-β1 (SMAD3 dependant) ↑	HDAC4 ↑, Cox-2 protein expression, NF-κB ↑
34c	HSC	↑	α-SMA ↑											PPARγ ↓
122	HSC	↓	collagen maturation and ECM production ↑					↑						P4HA1 ↓
130b	HSC		ECM ↑					↑						PPARy ↓
144	HSC	↓	α-SMA ↑								↑		TGF- β1 ↑	

**Table 3:** MiR families and clusters that have been identified as important in CAFs/HSCs/PSCs.

**miR family**	**8**		**15**			**17**			**27**		**29**		**33**	**34**		**101**	
**chromosomes**	12	1	13	3	17	13	7	x	9	19	7	1	17	1	11	1	9
**members of miR-clusters**	141 200c	200a 200b 429	15a, 16-1	15b, 16-2	195 497	17 18a 19a 19b-1 20a 92a-1	25 93 106b	18b 19b-2 20b 92a-2 106a 363	23b 24-1 27b 3074	23a 27a 24-2	29a 29b-1	29b-2 29c	33a 33b 6777	34a	34b 34c	101-1 3671	mir-101-precursor-9
																	
																	
**miR family**	122	127	130		133			143/ 145	144	154	181		214	221	342	365	
**chromosomes**	18	14	11	22	18	20	6	5	17	14	1	9	1	x	14	16	17
**members of miR-clusters**	122 3591	127 136 337 431 433 432 665	130a	130b 301b	1-2 133a-1	133a-2	133b 206	143 145	144 451a 451b 4732	134 154 323b 365 377 409 410 412 485 496 541 656	181a-1 181b-1	181a-2 181b-2	199a-2 214 3120	221 222	151b 342	193b 365a	365b 4725

## Supplementary Materials

The following are available online at http://www.mdpi.com/2072-6694/9/6/54/s1. Table S1: MiRs identified with specific roles in fibroblasts under pathological conditions (CAFs, HSCs, PSCs)OC (Ovarian Cancer), EC (Endometial Cancer), BC (Breast Cancer), GC (Gastric Cancer), EOC (Esophageal Squamous cell carcionoma), PaC (Pancreatic Carcinoma), CRC (Colorectal Cancer), PC (Prostate Cancer), LC (Lung Cancer).
