# Supplementary material for: Dysregulation of miRNA Expression in Cancer Associated Fibroblasts (CAFs) and Its Consequences on the Tumor Microenvironment
Source: Cancers (Basel). 2017 May 24;9(6):54. doi: 10.3390/cancers9060054 (PMC5483873; doi:10.3390/cancers9060054)
Supplement: Supplementary file 1 [file cancers-09-00054-s001.pdf]

# Supplementary Materials: Dysregulation of miRNA Expression in Cancer Associated Fibroblasts (CAFs) and Its Consequences on the Tumor Microenvironment

**Maren Schoepp, Anda Jana Ströse and Jörg Haier**

**Supplemental Table S1 MiRs identified with specific roles in fibroblasts under pathological conditions**

| Cell type | miRNA | Direction of deregulation | Type of validation | Functional effect in NFs turning to CAFs | Interaction in CAFs | Ref. |
|---|---|---|---|---|---|---|
| OC | 31<br>214<br>155 | ↓ | in vivo: paired samples of CAF (metastasis) and NF; in vitro: NF co-cultured with HeyA8 OC cells | proliferation ↑ | cytokines ↑<br>target: CCL5 | [5] |
| EC | 31 | ↓ | in vitro: paired samples of EC tissue and tumor free tissue; in vivo: mouse xenograft model | migration, invasion of NFs ↑ | target: SATB2 ↑ | [11] |
| | 148a | ↓ | in vitro: paired samples of EC tissue and tumor free tissue | | target: WNT10B | [10] |
| BC | 26b | ↓ | in vitro: paired samples of BC tissue and tumor free tissue culture models | ↓ | targets: TNKSIBPI, CPSF7, COL12A1 | [12] |
| | 31 | ↑ | in vitro: BC tissue compared with tumor free tissue | activation ↑ | $\alpha$-SMA, IL-6 ↑, pathway: TGF-ß ↑ | [14] |
| | **221** | ↑ | | | | |
| | **200b** | ↓ | | | | |
| | **200c** | ↓ | | | | |
| | **101** | ↓ | | | | |
| | **141** | ↓ | | | | |
| | **342** | ↓ | | | | |
| | 205,<br>let -7g,<br>26b | ↓ | | | | |
| | **127** | ↓ | in vitro: BC cell lines and sets of primary breast tumors and adjacent normal tissues from the same patients | proliferation ↑ | p53/p21 ↑<br>target: BCL6 oncogene ↑ | [46] |
| | 22 | ↑ | in vitro: epithelial and BC cell lines, in vivo: by inducing cellular senescence in a mouse model of breast carcinoma | proliferation ↓ | targets: CDK6, SIRT1, and Sp1 genes | [71] |
| | **92** | ↓ | in vitro: tissue samples and in breast epithelium and stroma during BC progression | expression of miR in FIBs ↓ | | [13] |
| | 146b | ↓ | in vitro and in vivo: tissue samples with and without BC | activation ↑ | IL-6 ↑<br>(p 16 related) | [41] |
| | **200** | ↓ | in vitro | activation ↑ | $\alpha$-SMA ↑<br>targets: Fli-1 is directly regulated by miR-200c, TCF12 is directly | [3] |

| **Cell type** | **miRNA** | **Direction of deregulation** | **Type of validation** | **Functional effect in NFs turning to CAFs** | **Interaction in CAFs** | **Ref.** |
|---|---|---|---|---|---|---|
| | | | | | targeted by miR-141 -> ECM remodeling ↑ | |
| | **365** | ↓ | in vitro, in vivo | ↓ | IL6 ↑ pathway: p38 MAPK ↓ target: NF-kBp65 ↑ | [63] |
| GC | **106b** | ↑ | in vitro: paired samples of GC tissue and tumor free tissue | proliferation ↑, motility ↑ | $\alpha$-SMA ↑, TGF-ß ↑ target: PTEN | [23] |
| | **200b** | ↓ | in vitro: gastric cancer cell lines compared with CAF; in vivo: mouse xenograft model; clinicopathological: comparing miR-200b expression in 173 patients | activation ↑ | $\alpha$-SMA ↑ targets: ZEB1, ZEB2 ↑ ; repressed E-cadherin expression | [15] |
| | 149 | ↓ | in vitro: GC cell lines, fibroblasts from tumor tissues and non-tumor tissues, in vivo: mouse xenograft model | activation ↑ | IL6 ↑ -> EMT (epithelial-to-mesenchymal transition) ↑ target: PGE2 induces the epigenetic silencing of miR-149 in fibroblasts | [79] |
| | **145** | ↑ | in vitro: tissue samples and cell lines | activation ↑ | $\alpha$-SMA ↑ pathway: TGF-ß ↑ | [25] |
| | **143** | ↑ | in vitro: tissue samples and cell lines, in situ hybridization | activation ↑ | Collagen type III ↑ pathway: TGF-β/SMAD signaling ↑ | [24] |
| EOC | **27a/b** | ↑ | in vitro: serum expression of miR, from tissue with and without cancer | activation ↑ | $\alpha$-SMA ↑ pathway: TGF-ß ↑ | [26] |
| | 21 | ↑ | in vitro: samples from patients with esophageal squamous-cell carcinoma, and a co-culture system of normal fibroblasts and esophageal cancer cells | activation ↑ | | [40] |
| PaC | 21 | ↑ | in vitro: tumor and tumor free tissue | activation ↑ | $\alpha$-SMA ↑ | [17] |
| | 21 | ↑ | in vitro: tumor and tumor free tissue | activation ↑ | | [17] |
| | 222 | ↑ | in vitro co-cultured experiments with: human normal and cancer associated stellate cells, human cancer associated fibroblast cells, human PaC cell lines | activation ↑ | $\alpha$-SMA ↑ | [4] |
| | **221** | | | | $\alpha$-SMA ↑, targets: NF-κB, K-Ras ↑ | |
| | 155 | ↑ | in vitro: isolated primary pancreatic fibroblasts from mice co-cultured with pancreatic cancer cell lines | activation ↑ (by microvesicles) | alpha SMA ↑ target: TP53INP1 ↓ by direct targeting of miR-155 and microvesicles | [65] |
| | **200a, 200b** | ↑ | in vitro: cell lines and tissue samples with and without cancer | activation ↑ | target: SIP1 by promoter methylation and retain expression of E-cadherin, regulating EMT | [16] |
| CRC | 21 | ↑ | in vitro: human cell lines and tissue samples with and without cancer | activation ↑ | cytokines ↑, PDCD4 ↓(inversely correlated with miR expression) | [27] |
| PC | 205 | ↓ | in vitro: cell culture | activation ↑ | inflammatory cytokine secretion ↓ | [45] |

| Cell type | miRNA | Direction of deregulation | Type of validation | Functional effect in NFs turning to CAFs | Interaction in CAFs | Ref. |
|---|---|---|---|---|---|---|
| | | | | | target: HIF-1 ↑, EMT ↑ | |
| | **409** | ↑ | in vitro: prostate and bone and in patient tissue samples and cells , in vivo: mouse xenograft | expression ↑ | α-SMA, EMT ↑, extracellular vesicle(EV) release ↑ | [19] |
| | **15**<br>**16** | ↓ | in vitro: 23 non-neoplastic and tumor tissues samples, in situ hybridization, in vivo: mouse model | ↓ | α-SMA ↑<br>targets: Fgf-2 and its receptor Fgfr1 | [20] |
| | **133b** | ↑ | in vitro: human PC cells and human prostate tissue with or without cancer | activation ↑ | α-SMA, IL-6 ↑ pathway: TGF-ß ↑ | [22] |
| LC | **101** | ↑ | in vitro: human lung cell lines, and human lung tissue with and without cancer | proliferation ↑ | pathway: PI3K-AKT ↓<br>targets: CXCL 12 ↓ | [21] |
| | **200b** | ↑ | in vitro: cell lines | activation ↑ | α-SMA ↑<br>target: Flt 1 ↓ | [42] |
| PSC | **143** | ↑ | Rat PSCs were isolated from the pancreas tissue of male Wistar rats. PSCs were activated in vitro by culture in serum-containing medium | activation ↑ | interaction with p39 mitogen-activated protein kinase, extracellular-signal–regulated kinase, and SMAD 2/4 pathway | [74] |
| | **221** | ↑ | | | interaction with p40 mitogen-activated protein kinase, extracellular-signal–regulated kinase, and SMAD 2/5 pathway | |
| | 31 | ↑ | | | interaction with p38 mitogen-activated protein kinase, extracellular-signal–regulated kinase, SMAD 2/3 pathway | |
| | 126 | ↓ | | | interaction with p41 mitogen-activated protein kinase, extracellular-signal–regulated kinase, SMAD 2/6 pathway | |
| | 146a | ↓ | | | interaction with p42 mitogen-activated protein kinase, extracellular-signal–regulated kinase, SMAD 2/7 pathway | |
| | 150 | ↓ | | | interaction with p43 mitogen-activated protein kinase, extracellular-signal–regulated kinase, SMAD 2/8 pathway | |
| | 210 | ↑ | in vitro: Panceatic cancer cells mono-cultured or indirectly co-cultured with PSCs | activation ↑ | EMT ↑<br>pathway: ERK and Akt ↑ | [61] |

| Cell type | miRNA | Direction of deregulation | Type of validation | Functional effect in NFs turning to CAFs | Interaction in CAFs | Ref. |
|---|---|---|---|---|---|---|
| | **15b** **16** | ↓ | in vitro: rat PSC | activation ↑ | target: BCL-2 ↑ | [66] |
| | **29** | ↓ | in vitro: Immortalized mouse pancreatic stellate (mPSC) cell lines and tissue samples, in vivo: mouse model | activation ↑ | ECM ↑ pathway: TGF-β1 (SMAD3 dependent) ↑ | [57] |
| HSC | 335 | ↓ | in vitro: cultured HSC from rats | activation ↑ | α-SMA and Collagen Type 1 ↑ target: TNC (tenascin-C) expression ↓ | [51] |
| | 150 | ↑ | in vitro: HSC isolated from sham-operated and bile duct-ligated rats compared with HSC isolated from fibrotic rats | activation ↓ | α-SMA and Collagen Type 1 ↓ target: C-myb ↓ | [56] |
| | 194 | ↑ | | | target: Rac 1 ↓ | |
| | 146a | ↓ | in vitro | activation ↑ | α-SMA and Col-1 ↑ targets: Wnt1 and Wnt5a ↑ | [59] |
| | 146a | ↓ | in vitro: cell culture, in vivo: rat model | activation ↑ | α-SMA ↑, TGF-ß ↑ by direct targeting of SMAD4 | [68] |
| | **200a** | ↓ | in vitro: cell culture, in vivo: rat model | activation ↑ | α-SMA ↑ pathway: Wnt/β-catenin and TGFβ ↑ | [47] |
| | **200a** | ↑ | no information | activation ↓ | α-SMA ↓, EMT process ↓, Gli2 (downstream signaling protein of the Hh pathway) ↓ | [81] |
| | **9a** | ↑ | in vitro: cell culture, in vivo: rsat model | activation ↑ | target: SIRT1 | [72] |
| | **17** | ↑ | no information | activation ↑ | Collagen Type I and α-SMA ↑, TGF-ß 1 ↑, SMAD7 ↓ (direct target) | [38] |
| | 33a | ↑ | in vitro: cell culture and human liver and serum samples | activation ↑ | α1 Collagen (Col1A1) and α-SMA ↑, TGF-ß1 ↑, PI3K/Akt pathway target: PPAR-α | [30] |
| | **200b** | ↑ | in vitro: human hepatic stellate cell line | proliferation, migration ↑ | PI3K/Akt through FOG2 ↓-regulation | [31] |
| | 21 | ↑ | in vitro: human immortalized HSC line | activation ↑ | Collagen Type I and α-SMA ↑, PTEN/Akt pathway | [54] |
| | 130a/b | ↑ | in vitro: immortalized rat HSC line, in vivo: rat model | activation ↑ | ECM ↑ target: PPARγ ↓ | [70] |
| | 30, 193 | ↓ | in vitro: hepatic fibrosis and human tissue samples, in vivo: mouse model | activation ↑ | TGF-ß , TGF-β2 and SNAIL1 as potential tagets | [34] |
| | **144** | ↓ | in vitro: human tissue samples | activation ↑ | α-SMA ↑, TGF-ß1 ↑ | [36] |
| | 146a | ↓ | in vivo: cell culture | activation ↑ | TGF-ß1 ↑ target: SMDA4 | [68] |

| Cell type | miRNA | Direction of deregulation | Type of validation | Functional effect in NFs turning to CAFs | Interaction in CAFs | Ref. |
|---|---|---|---|---|---|---|
| | 126 | ↑ | no information | activation ↑ | TGF-ß1, IκBα protein expression ↓ pathway: NF-κB | [44] |
| | **221 222** | ↑ | in vitro: cell culture and liver biopsy specimens, in vivo: mouse model | activation ↑ | α1-Collagen and α-SMA ↑ | [32] |
| | **19b (-1/-2)** | ↓ | in vitro: rat HSCs, in vivo: rat model | activation ↑ | Type I Collagen ↑, expression of α1(I) and α2(I) procollagen in mRNAs ↑ pathway: TGF-ß ↑ targets: TGF-ß 2 receptor and SMAD 3 | [33] |
| | **101** | ↑ | in vitro: Primary rat and mouse HSCs, in vivo: mouse model | activation ↓ | targets: TβRI and KLF6, inhibiting TGFβ signalling pathway | [49] |
| | 31 | ↑ | in vitro: human tissue samples, in vivo: rat model | activation ↑ | TGF-ß ↑ | [52] |
| | **19b (19b-1, 19b-2)** | ↑ | in vitro: tissue samples, in vivo: rat model | activation ↓ | target: GRB2 | [64] |
| | 34a/ | ↑ | in vitro: human HSC line, in vivo: rat model | activation ↑ | α-SMA ↑ target: PPARγ ↓ | [69] |
| | 21 | ↑ | in vitro: human tissue samples, rat HSC cell line | activation ↑ | induces hepatocyte EMT pathway: ERK1 targets: SPRY2, HNF4α | [28] |
| | **101** | ↑ | in vitro: Primary rat and mouse HSCs, in vivo: mouse model | activation ↓ | TGFß signaling ↓ by suppressing TβRI/KLF6 (targets) | [49] |
| | 181 b | ↑ | in vitro: human cell line, in vivo: rat model | activation ↑ | α-SMA and Type I Collagen ↑ pathway: PI3K/Akt ↑ target: PTEN expression ↓ | [54] |
| | **200a** | ↑ | in vitro: HSC-T6 cell lines, in vivo: rat model | activation ↓ | pathway/ targets: Keap1/Nrf2; Nrf2 ↑ inhibits TGF-β1 induced growth | [73] |
| | 483 | ↑ | in vitro: cell lines, in vivo: mouse model | activation ↓ | TGF-ß ↓ targets: TIMP-2 and PDGF-β ↓ | [62] |
| | 21 | ↑ | in vivo: mouse model | activation ↑ | TGF-ß, α-SMA ↑ targets: PDCD4/AP-1 ↑ | [29] |
| | 126 | ↓ | in vitro: primary rat HSC, in vivo: rat model | activation ↑ | PI3K, p-AKT ↑ | 82 |
| | **29a** | ↓ | in vitro: mice HSC, in vivo: mouse model | activation ↑ | alpha SMA ↑ target: HDAC4 ↑ | [58] |
| | 150 | ↑ | in vitro: human cell lines | activation ↓ | alpha SMA ↓ targets: Sp1 and Col4A4 | [83] |
| | 133a | ↓ | in vitro: cell lines, in vivo: mouse model | activation ↑ | TGF-ß, collagen ↑ | [35] |
| HSC | **122** | ↓ | in vitro: rat HSC, in vivo: rat model | activation ↑ | collagen maturation and ECM production ↑ | [50] |

| Cell type | miRNA | Direction of deregulation | Type of validation | Functional effect in NFs turning to CAFs | Interaction in CAFs | Ref. |
|---|---|---|---|---|---|---|
| | | | | | target: P4HA1 ↓, binding activity of C/EBPα to miR-122 promoter | |
| HSC | 181b | ↑ | in vitro: serum samples and human cell lines | activation ↑ | TGF-ß ↑<br>target: p27 | [39] |
| HSC | 26a | ↓ | in vitro: primary rat HSC, in vivo: rat | activation ↑ | Collagen Type I , Cox-2 protein expression, NF-κB ↑ | [60] |
| | **29a** | ↑ | | | | |
| | **214** | ↓ | | | | |
| | 146a | ↓ | | | | |
| HSC | 199a, 199a* | ↑ | in vitro: human clinical samples, in vivo: mouse model | activation ↑ | TGF-ß ↑ | [37] |
| | **200a, 200b** | ↑ | | | | |
| HSC | 16 | ↓ | in vitro: rat HSC | activation ↑ | CD1, Bcl-2 ↑ | [67] |
| HSC | **27a/b** | ↑ | in vitro: rat HSC | activation ↑ | target: retinoid X receptor alpha | [84] |

(CAFs, HSCs, PSCs) OC (Ovarian Cancer), EC (Endometial Cancer), BC (Breast Cancer), GC (Gastric Cancer), EOC (Esophageal Squamous cell carcionoma), PaC (Pancreatic Carcinoma), CRC (Colorectal Cancer), PC (Prostate Cancer), LC (Lung Cancer).

## References


75. Baroni, S.; Romero-Cordoba, S.; Plantamura, I.; Dugo, M.; D'Ippolito, E.; Cataldo, A.; Cosentino, G.; Angeloni, V.; Rossini, A.; Daidone, M.; et al. Exosome-mediated delivery of miR-9 induces cancer-associated fibroblast-like properties in human breast fibroblasts. *Cell Death Dis.* **2016**, *7*, e2312, doi:10.1038/cddis.2016.224.
76. Nouraee, N.; Khazaei, S.; Vasei, M.; Razavipour, S.F.; Sadeghizadeh, M.; Mowla, S.J. MicroRNAs contribution in tumor microenvironment of esophageal cancer. *Cancer Biomark.* **2016**, *16*, 367–376.
77. Takikawa, T.; Masamune, A.; Yoshida, N.; Hamada, S.; Kogure, T.; Shimosegawa, T. Exosomes Derived from Pancreatic Stellate Cells: MicroRNA Signature and Effects on Pancreatic Cancer Cells. *Pancreas* **2017**, *46*, 19–27.
79. Au Yeung, C.L.; Co, N.N.; Tsuruga, T.; Yeung, T.L.; Kwan, S.Y.; Leung, C.S.; Li, Y.; Lu, E.S.; Kwan, K.; Wong, K.K.; et al. Exosomal transfer of stroma-derived miR21 confers paclitaxel resistance in ovarian cancer cells through targeting APAF1. *Nat. Commun.* **2016**, *7*, 11150.
80. Li, P.; Shan, J.X.; Chen, X.H.; Zhang, D.; Su, L.P.; Huang, X.Y.; Yu, B.Q.; Zhi, Q.M.; Li, C.L.; Wang, Y.Q.; et al. Epigenetic silencing of microRNA-149 in cancer-associated fibroblasts mediates prostaglandin E2/interleukin-6 signaling in the tumor microenvironment. *Cell. Res.* **2015**, *25*, 588–603.
81. Yu, F.; Zheng, Y.; Hong, W.; Chen, B.; Dong, P.; Zheng, J. MicroRNA 200a suppresses epithelial to mesenchymal transition in rat hepatic stellate cells via GLI family zinc finger 2. *Mol. Med. Rep.* **2015**, *12*, 8121–8128.
82. Guo, C.J.; Pan, Q.; Xiong, H.; Qiao, Y.Q.; Bian, Z.L.; Zhong, W.; Sheng, L.; Li, H.; Shen, L.; Hua, J.; et al. Dynamic expression of miR-126* and its effects on proliferation and contraction of hepatic stellate cells. *FEBS Lett.* **2013**, *29*, 3792–3801.
83. Zheng, J.; Lin, Z.; Dong, P.; Lu, Z.; Gao, S.; Chen, X.; Wu, C.; Yu, F.; et al. Activation of hepatic stellate cells is suppressed by microRNA-150. *Int. Jo. Mol. Med.* **2013**, *32*, 17–24.
84. Ji, J.; Zhang, J.; Huang, G.; Qian, J.; Wang, X.; Mei, S. Over-expressed microRNA-27a and 27b influence fat accumulation and cell proliferation during rat hepatic stellate cell activation. *FEBS Lett.* **2009**, *583*, 759–766.
